# Evidence for the effectiveness of digital health

**DOI:** 10.1038/s41746-020-0231-9

**Published:** 2020-03-10

**Authors:** Joseph C. Kvedar

**Affiliations:** Partners Healthcare, Harvard Medical School, Boston, MA USA

Twenty-five years ago, I participated in a research study at Massachusetts General Hospital to examine the feasibility of adopting a new type of technology called digital imaging. We hoped this new technology would be of sufficient quality to allow a dermatologist to diagnose skin diseases from afar, and that project changed my life. Amid the dry analysis of diagnostic concordance, I had an epiphany, realizing that the opportunity to use digital technologies to change the way we provide healthcare fundamentally, was vast and breathtaking. The vision of healthcare delivered directly to wherever the patient is located around the globe, and precisely when the patient needs the care, was born then, and is alive and well today.

In the ensuing years, we have made great strides towards integrating digital technologies into the clinic, but we still have far to go. For example, we now take for granted that the core technology for digital imaging in dermatology is of diagnostic quality. Clinicians routinely capture images with commercial-grade (i.e., smartphone) digital cameras and use them to break the barriers of time and space to improve care delivery. However, this model of care delivery is by no means mainstream, and there are still plenty of skeptics challenging its veracity. Are digital images of sufficient quality to allow us to diagnose pigmented lesions for all skin tones? By reducing our physical exam to captured images, will we miss critical diagnostic clues? Does providing consultative advice directly to a primary care colleague—and never seeing the patient in-person—result in quality care, as compared to an in-person evaluation? These and additional questions continue to abound.

I am delighted to point out that these are all empiric questions. Virtually all of the controversies surrounding the growth and mainstreaming of digital medicine are empiric. That is what makes my new role as Editor-in-Chief for this critical journal such an exciting opportunity.

In 2016, the American Medical Association surveyed their membership regarding physician reticence to adopt digital medicine.^1^ Their research revealed four chief barriers to adoption, phrased as questions: Does it work (i.e., what is the evidence)? will I get paid? will I get sued, and can we integrate it into my practice? The first of these barriers was the natural skepticism that any physician shows when confronted with a new way of providing or enhancing care: Does it Work (i.e., what is the evidence base)?

This questioning of any innovation is natural and healthy. The philosophy of science hinges on the power of deductive reasoning and methodologically sound inquiry. Yet, we can see examples of tools that have overcome this natural skepticism. We do not ask “does the stethoscope work?” When Laennec introduced this tool, it took more than 20 years to reach widespread adoption.^2^ The first pioneers doing laparoscopic surgery were shunned by their peers, whereas now, we consider those who do not use this technique as committing malpractice. In both cases, a critical component of achieving mainstream adoption was the gathering of sufficient evidence to demonstrate clinical efficacy.

If we look at digital medicine in the context of Everett Rogers’ seminal work, The Diffusion of Innovations,^3^ we are still mainly in the early adopter phase, struggling to get into the early majority phase. One critical piece moving us along the curve is the accumulation of high-quality evidence, and there is no better way to curate evidence than through investigative inquiry published in high-quality peer-reviewed settings.

It is this backdrop that fuels my excitement in joining the team at npj Digital Medicine. Working with a talented editorial staff and an extraordinary team of associate editors, my life as Editor-in-Chief should easy! I look forward to my role in growing the evidence base that will enable us to integrate digital medicine into care delivery in a way that improves access and quality.

At some point soon, I hope, because of the work featured in this journal, the need to differentiate “digital” medicine will cease, as we see digital tools seamlessly integrated into the practice of “medicine”.
